# High levels of used syringe use and unsafe sex among people who inject drugs in Kumasi, Ghana: an urgent call for a comprehensive harm reduction approach

**DOI:** 10.1186/s12954-021-00510-7

**Published:** 2021-06-10

**Authors:** Lisa J. Messersmith, Rose Adjei, Jennifer Beard, Angela R. Bazzi, Joel J. Earlywine, Edwin Darko, Thomas Agyarko-Poku, Mabel Kissiwah Asafo, Sherry Adoma Bempah, Yaw Adu-Sarkodie

**Affiliations:** 1 Department of Global Health, Boston University School of Public Health, 801 Massachusetts Ave., Boston, MA 02118 USA; 2 Kwame Nkrumah University of Science and Technology School of Medicine, Kumasi, Ghana; 3 Herbert Wertheim School of Public Health and Human Longevity Science, University of California, San Diego, La Jolla, CA USA; 4 Department of Health Services, University of Washington, Seattle, WA 98195 USA; 5 Ghana Health Service, Kumasi, Ghana; 6 Institute for Distance Learning, Kwame Nkrumah University of Science and Technology, Kumasi, Ghana

**Keywords:** HIV, Injecting drug use, Needle sharing, Unsafe sex, Harm reduction, Ghana

## Abstract

**Background:**

Drug use is a growing concern in Ghana. People who inject drugs (PWID) are highly vulnerable to HIV and other infectious diseases. Ghana’s National Strategic Plan for HIV/AIDS 2016–2020 identifies PWID as a key population, but efforts to address the needs of PWID have lagged behind those targeting sex workers and men who have sex with men. Lack of information about PWID is a critical barrier to implementing effective HIV prevention and treatment. We aimed to learn more about the vulnerability of the PWID population in order to inform much-needed harm reduction interventions.

**Methods:**

From April to July 2018, we conducted a mixed methods study in Kumasi, Ghana, to identify all major drug using locations, count the numbers of PWID to obtain rough population size estimations, and administer anonymous surveys to 221 PWID regarding drug use and sexual behavior. We also tested for HIV, HCV, and HBV from syringes used by survey participants.

**Results:**

Key informants identified five major drug using locations and estimated the total PWID population size to be between 600 and 2000. Enumerators counted between 35 and 61 individuals present at each of the five bases. Sharing syringes and reusing discarded syringes are common practices. Over half of survey participants (59%) reported past-month syringe sharing (34% used a used syringe and 52% gave away a used syringe). Individuals with higher injection frequency (≥ 21 times weekly) and who injected with four or more people had higher odds of syringe sharing. Of the survey participants reporting sex in the last month (23%), most reported having one partner, but only 12% used condoms. Nearly all women (11/13) reported exchanging sex for drugs and 6/13 reported exchanging sex for money in the last six months. Fifteen percent of participants (all men) reported paying for sex using drugs or money. Of the used syringes, prevalence estimates were 3% (HIV), 2% (HCV), and 9% (HBV).

**Conclusions:**

Our findings confirm the urgent need to implement harm reduction interventions targeting PWID and to build a strong and enabling legal and policy environment in Ghana to support these efforts.

**Supplementary Information:**

The online version contains supplementary material available at 10.1186/s12954-021-00510-7.

## Background

In March 2020, the Government of Ghana signed the Narcotics Control Commission Bill, into law [1, 2], marking a historic shift toward treating drug use and addiction as a public health challenge rather than a purely criminal justice problem [3]. People who inject drugs (PWID) are highly vulnerable to HIV and other infectious diseases. The Ghana AIDS Commission, which oversees HIV research, programs, and advocacy, has devoted considerable resources toward addressing HIV vulnerability among key populations at risk of HIV infection, but to our knowledge no harm reduction programs have been implemented for PWID. While adult HIV prevalence in Ghana is estimated at 2% overall, prevalence is much higher among female sex workers and men who have sex with men (~ 13% and 18%, respectively) [4–6]. Ghana’s National Strategic Plan for HIV/AIDS 2016–2020 identifies PWID as another key population, but notes that the current PWID population size and HIV prevalence are unknown [7].

Research focused on other highly vulnerable populations provides some insight into potential HIV transmission from injection drug use. Yet, there is little information on the prevalence of injection drug use in Ghana or of blood-borne infections among PWID. A 2008 prison-based study found that of the 35.2% of 1399 prisoners who reported ever having injected drugs, the odds ratios of testing positive for HIV, HBV, and HCV were 5.7, 5.4, and 5.3 respectively. Of those who reported sharing needles or injection implements (71.5%), the odds ratios for testing positive for HIV, HBV, and HCV were 2.0, 1.9, and 1.9, respectively. Risk factors for these infections included unprotected sex, sharing of needles and injection equipment for drug use, and sharing of needles and ink for tattooing [8]. A 2009 study of female sex workers found that 7.4% of their clients and non-paying sex partners had past-year heroin use [5]. While these studies suggest that injection drug use occurs in key populations in Ghana, research characterizing the prevalence of injection drug use and specific HIV-related risk behaviors among PWID in Ghana is lacking. This research gap limits policymakers’ abilities to develop adequate prevention programs for this vulnerable and understudied population.

Kumasi is Ghana’s second largest city and an inland economic hub. Along with Accra, the capital, Kumasi is a primary destination of migrants from rural areas and has been the focus of HIV prevention programming for key populations. In 2013–2014, we conducted a small qualitative study focused on HIV knowledge, risk behaviors, perceived vulnerabilities, and health care needs [9]. Health service utilization was low and most respondents wanted better access to harm reduction and treatment services.

To build on this work, we conducted a more comprehensive mixed methods study of PWID behaviors and prevention needs across Kumasi. Our findings provide a novel snapshot of infectious disease risk among PWID throughout Kumasi that can inform the development of prevention services for this population.

## Methods

### Study design and population

From April to July 2018, we conducted a four-part mixed methods study. First, we conducted qualitative interviews with PWID who helped us identify all major “bases” in Kumasi (i.e., public areas where drugs are purchased and injected; also known locally as “ghettos”). These informants also facilitated our access to bases for subsequent data collection. Second, we visited major bases to count the numbers of individuals present (i.e., PWID enumeration). Third, we administered anonymous surveys to PWID present at bases. Fourth, we collected and tested used syringes from survey participants for the presence of HIV, HCV, and HBV (i.e., prevalence estimation).

For the key informant interviews, we recruited PWID who participated in our previous study who were purposively selected based on their knowledge about injecting drug use and the local bases. Qualitative interview and survey participants were eligible if they were ≥ 18 years old, currently injecting drugs (i.e., injected in the last two weeks and had physical evidence of injecting), living in Kumasi, and fluent in Twi or English. All participants provided verbal informed consent. Institutional Review Boards of Kwame Nkrumah University of Science and Technology (KNUST) and Boston University Medical Campus approved all study protocols.

### Data collection

For key informant interviews, trained interviewers used semi-structured interview guides with open-ended questions designed to explore the size and key features of Kumasi’s PWID population and areas (typically referred to as bases) where people purchase and use drugs. Qualitative interviews were conducted in private rooms at the major hospital and audio-recorded for transcription. Data collectors conducted interviews in Twi while simultaneously taking notes in English. This is standard practice in Ghana.

For PWID enumeration, 2–5 research team members visited each major base and counted all individuals present (who we assumed to be PWID) during a 4–6 h period between 8:30 am and 10:00 pm (depending on peak hours at each base).

Following enumeration, research team members also administered anonymous surveys to 221 PWID at these bases to determine *sociodemographics* (age, gender, educational level, marital status, ethnicity, religion, occupation, income, housing), *sexual behaviors* (condom use, numbers of partners, engagement in buying and selling sex), *drug injection behaviors* (syringe source, number of PWID known in the community, places where PWID inject drugs, frequency of injections, past-month sharing of injection equipment or syringes [i.e., using and/or giving away a used syringe], types of drug injected), *HIV testing*, and *healthcare and prevention service access and needs*.

For prevalence estimation, from each survey participant, we collected one used syringe (and provided three new ones). We used this method of testing for blood-borne infections because we did not have the resources to collect and test blood or saliva. Used syringes were labeled with participants’ identification numbers and tested for HIV, HCV, and HBV. Serological testing for HIV, HCV, and HBV antibodies used Ghana Ministry of Health (MOH)-approved assays and protocols [10]. Testing was conducted at the Serology Laboratory of the Komfo Anokye Teaching Hospital (SL-KATH) in Kumasi. For HIV testing, SL-KATH scientists flushed residual blood out of sets using a manufacturer-supplied buffer for the SD Bioline HIV rapid test (SD Bioline, Korea) and tested 10 µl of the solution for HIV-1 and HIV-2 antibodies according to manufacturer instructions. All HIV positive samples were confirmed using Oraquick rapid tests (OraSure Technologies Inc, USA). They also tested 10 µl of solution for HCV and HBV antibodies according to manufacturer instructions (SD Bioline, South Korea).

### Data analyses

Researchers coded and analyzed the data focusing on behaviors, including syringe sharing and condom use. To enumerate PWID, we calculated averages of research team members’ counts of individuals present at the bases. With survey data, we calculated descriptive statistics to summarize participant characteristics and compare those who did and did not engage in past-month syringe sharing (i.e., using and/or giving away a used syringe). One participant was excluded due to missing data on key variables of interest. Bivariable logistic regression with standard errors clustered by base identified factors associated with syringe sharing. To build a final, multivariable model identifying factors independently associated with syringe sharing, we entered all variables attaining significance (*p* < 0.10) in bivariable analyses into a full model and assessed collinearity using a variance inflation factor (VIF; cutoff: 4) [11]. When removing nonsignificant variables from the full model, we assessed for the presence of confounding via changes in other estimates of ≥ 10% [12] and compared the fit of nested models using AIC [13]. We included all variables retaining significance at the *p* < 0.05 level as well as those identified as potential confounders in the final multivariable model.

For HIV, HCV and HBV prevalence estimation, we summarized the laboratory testing results overall and by base. Finally, we integrated qualitative and quantitative data (including descriptive statistics) on health, drug treatment, and harm reduction service needs.

## Results

### Size and key features of Kumasi’s PWID population

Key informants (*n* = 7) identified five major bases across Kumasi where they believed most PWID purchased and injected drugs. They described bases as abandoned buildings and a cemetery that were infrequently visited by the public. They estimated the numbers of PWID routinely attending these bases to range from 25 to over 1000, depending on the base. They agreed that Base 4 was the largest, with between 350 to over 1000 unique individuals routinely injecting drugs there (three informants estimated the lower end of this range at 700). Across all five bases, key informants estimated the total PWID population size in Kumasi to be 600 to over 2000. On average, key informants personally knew more than 15 PWID who were mostly men. When researchers visited bases for PWID enumeration, average counts were between 35 and 61 individuals present during their visits (Table 1).

**Table 1:** Numbers of PWID present at one time at the five major “bases” in Kumasi, Ghana, 2018

Date of enumeration	Base	Average # of PWID present^a^
May 3, 2018	Base 1	39
May 9, 2018	Base 2	35
June 18, 2018	Base 3	57
June 19, 2018	Base 4	61
June 27, 2018	Base 5	46

^a^Average of 2–5 enumerators’ counts of PWID present at each base

### PWID characteristics and behaviors

#### Sociodemographics

Among PWID who completed anonymous surveys (*n* = 221), median age was 34 years (interquartile range [IQR]: 29–41 years) and most were male (94%), Akan ethnicity (89%), Christian (83%), and never married (64%; Additional file 1: Table X). Half had completed primary school (50%). Most worked as laborers or porters and median weekly income was 150 Ghana cedis (USD $28; IQR: 100–200 cedis). Almost half lived with one or more friends or coworkers (46%), and one third lived in households of four or five people (37%).

#### Sexual behaviors

About a quarter of survey participants (23%) reported having sex in the last month. Of those, most reported having one partner, and only 12% used condoms. Nearly all women (11 out of 13) reported exchanging sex for drugs in the last six months, and 6 out of 13 reported exchanging sex for money. Fifteen percent of participants (all men) reported paying for sex using drugs or money. Only 41 participants (19%) had an intimate partner with whom most (95%) did not use condoms. Of those who had an intimate partner, 12% (*n* = 5) reported injecting drugs with their partner and either always or sometimes sharing syringes.

As illustrated in the quotes below, all seven qualitative key informants described how it was very common for women to exchange sex for money or drugs, often at the insistence of male partners who also used drugs.

“As for the women, [sex work] is their usual work they do. When she needs some drugs to inject, they usually will sleep around for money.”

“A lot of the women [have sex for money]. [Q: What of the men?] The men don’t sell sex, what they usually do is to steal.”

“Some go and do prostitution to get money so she can get money for her boyfriend to shoot. Some are beaten to go and do so. [Q: So do the men and women do prostitution for money?] That’s what goes on there.”

#### Drug injection behaviors

All participants reported injecting heroin, with about half injecting 2–5 times daily (46%) or 6–10 times daily (47%). Most injected with other PWID at the same time (71%), with 44% injecting with an average of 4–5 other people, and 42% with an average of ≥ 6 other people. Most participants injected at bases (including abandoned buildings), at home, or under a bridge.

Like our key informants, survey participants reported knowing a median of 15 other PWID (IQR:15–20). Key informants described sharing syringes and using used or discarded syringes as a common practice among PWID in Kumasi, especially among those who could not afford purchasing new ones in pharmacies.

“We usually use new needles but some people who cannot afford will end up using the ones I have used already. […] Most people don’t check the neatness [cleanliness] of the syringe]. Once I have used it and he needs it, he just comes for it.”

“We buy the assorted [drugs] and mix it, then we all pull from the same source. It’s expensive so it’s okay to contribute with colleagues [to] then buy and share.”

“Some people don’t mind using other people’s needle because their own may be spoilt. Others wouldn’t mind giving out their needles because they may get some of the drug left over in the needle. Some people, no matter the relation they have with you, will never give out their needles. They use it alone no matter what.

While several participants said they did not share needles, their statements were often qualified with acknowledgement that they share with their close friends and/or intimate partners.

“Because we all know that the HIV, you can get it using metallic sharp objects, so they don’t share needles unless you’re his best friend. Me, for instance, I won’t share my needles. I share with only my girlfriend.”

One informant also explained that some PWID have connections to hospitals or clinics where they obtain syringes for personal use or to sell at the bases. Other informants explained that PWID often use syringes they find on the “floor.” When asked if those syringes look “neat” or “old and rusty,” the informant replied, “*No. the needles are not expensive. You see people who are so gentle and neat. Others have gone mad due to shooting. They go around picking the disposed-of needles.”*

Almost all survey participants (96%) reported obtaining syringes from friends or purchasing them from pharmacies at an average price of 1 Ghana cedi (USD $0.19) per syringe. Over half (59%) reported past-month syringe sharing (34% used a used syringe and 52% gave away a used syringe to others in this time period). In our final multivariable model accounting for recruitment base, sociodemographics (age, ethnicity, occupation), and reported numbers of drug users and places where people inject drugs in the community, individuals with higher injection frequency (≥ 21 times weekly) and who injected with four or more people had higher odds of syringe sharing (Table 2). Of note, those who reported that they would use a syringe exchange program, if available, also had higher odds of syringe sharing than those who did not believe they would use such a service.

**Table 2:** Factors independently associated with past-month syringe sharing among PWID completing surveys at major bases in Kumasi, Ghana, 2018 (*n* = 221)^a^

Variable	Adjusted Odds Ratio	95% CI
Injection frequency		
1–20 times a week	*Reference*	
21 or more times a week	3.76	2.01–7.04
How many people do you inject with?		
0–3 people	*Reference*	
4 or more people	2.43	1.10–5.38
Would you use a syringe exchange program?		
Yes	3.53	1.70–7.31
No	*Reference*	

^a^Final regression model includes standard errors clustered by base and controls for sociodemographic characteristics (age, ethnicity, occupation, number of drug users in the community, and number of places where people inject drugs)

### HIV, HCV, and HBV prevalence

Of the used syringes we collected, HIV was present in six (5 men, 1 woman), HCV was present in five (all men), and HBV was present in 19 (18 men, 1 woman), yielding prevalence estimates of 3% (HIV), 2% (HCV), and 9% (HBV; Table 3). Base 3 had the highest numbers of syringes testing positive for these infections (*n* = 13), followed by Base 2 (*n* = 7).

**Table 3:** HIV, HCV, and HBV in syringes provided by PWID completing surveys at major bases in Kumasi, Ghana, 2018 (*n* = 221)

Infection	Base 1 (*n* = 44)	Base 2 (*n* = 65)	Base 3 (*n* = 72)	Base 4 (*n* = 16)	Base 5 (*n* = 24)	Overall (*n* = 221)
HIV	0 (0%)	1 (2%)	4 (6%)	0 (0%)	1 (4%)	6 (3%)
HCV	1 (2%)	0 (0%)	1 (1%)	2 (13%)	1 (4%)	5 (2%)
HBV	3 (7%)	6 (9%)	8 (11%)	2 (13%)	0 (0%)	19 (9%)

### Prevention needs

Only 35% of participants had ever been tested for HIV (and only 2 had HIV testing in the past six months). No participants had been tested for HCV; only one had been tested for HBV. Most (92%) believed that PWID experience stigma while receiving healthcare services.

In qualitative interviews, all seven key informants said their main health need was to stop using drugs and wanted treatment centers to include medications for opioid use disorder (e.g., methadone) to help “*bring down that crave we have for shooting.”*

“We need you to help us stop this lifestyle. You should assist us stop […] so they should open a rehabilitation centre. People are dying.”

“Some people shoot but they don’t bathe and have tattered clothes. They use all their monies for drugs. So the help is to get them back in good clothes and get medication that would help bring down that urge to go and shoot.”

Informants also requested housing resources, as one explained, “*a place to house us and give us beds; if you’re there and you realize the life you’re living isn’t the best, you can stop the lifestyle.”* Another informant also believed that “*when we are housed and we have a place to sleep and bath, you can personally start cutting down on the number [of drugs used].”*

Most key informants also thought syringe exchange programs would be useful, with one stating, “*If I haven’t stopped the lifestyle of drugs and you want to change my needles for me, I would like that help*.” Another said it would be helpful to have a greater “*supply of needles so that we don’t get infected”* and “*so that we don’t go sharing needles to make us ill.”*

Among survey participants, 81% said they had access to general health care and only 6% to addiction recovery treatment. When asked what new services they would use if available, 96% of participants said they would use addiction treatment; 74% would use a drop-in center; 43% would use syringe exchange programs, and 23% said stigma-reduction services were needed in their community.

## Discussion

Ghana sits directly in the path of the global drug trade, with heroin passing through cities in West Africa as it moves from Asia to North America [14, 15]. Some trafficked drugs remain in Ghana, contributing to addiction and threatening progress made in HIV prevention over the last decade. While Ghana has done much to address HIV among sex workers and men who have sex with men, fewer political and economic resources have been dedicated to HIV prevention and other health services (including addiction treatment) for PWID. In our previous research, we documented profound vulnerability and HIV risk in a small sample of PWID in Kumasi. While these findings were of great interest to the Ghana AIDS Commission, development partners, and non-governmental organizations, there was reluctance to fund harm reduction efforts in the absence of data on the size and characteristics of the larger PWID population [16].

In the context of drug policy reforms and focused HIV prevention efforts on key populations, we sought to contribute data to inform efforts to better engage PWID in evidence-based HIV prevention strategies and health services. Programmatic efforts require knowledge of the local PWID population size [17], which we estimate to be between a few hundred to two thousand individuals across the five largest bases in Kumasi (i.e., excluding individuals who attend smaller venues). We observed consistent estimations across key informants regarding the total number of PWID in Kumasi to be in the thousands. However, future studies using more formal methods (e.g., capture-recapture design) will be needed to confirm these estimates [18–20].

Access to sterile syringes and injection equipment within the major bases in Kumasi was severely limited. We found high acceptability for syringe exchange programs in our sample. As found elsewhere, those who reported that they would use a syringe exchange program, if available, also had higher odds of syringe sharing than those who did not believe they would use this service [21–24]. This finding suggests that PWID who share syringes recognize their risk and will therefore benefit from harm reduction efforts.

Although our HIV, HCV, and HBV syringe testing suggested low prevalence of these infections, this study provides the first estimates of HIV prevalence among PWID in over a decade and the first ever—as far as we know—of HBV and HCV prevalence to be published. Adjei et al. found that more than one third of prisoners in Ghana had ever injected drugs (11.5% of whom were HIV positive), and that a history of injecting drug use and syringe sharing were positively associated with HIV infection [8].

A 2020 systematic review and meta-analysis of HBV prevalence in Ghana found that HBV prevalence varied by region, with higher levels in the south, and was highest among special occupations such as barbers and long-distance truck drivers (14.4%) and lowest among blood donors (7.1%) [25]. A recent systematic review and meta-analysis to estimate HCV prevalence in Ghana found the national prevalence of chronic HCV infection to be 3%, with higher levels in rural than urban areas (5.7% vs. 2.6%) [26]. Neither study identified PWID specifically.

Treatment programs for substance use disorders are not readily available [27]. Drug treatment is provided by psychiatric hospitals, some regional hospitals, and some non-governmental and faith-based organizations, almost all of which are located in the southern part of the Ghana (Accra-Tema, Kumasi, and Sekondi-Takoradi). None provide medication-assisted treatment, peer education, or injection equipment exchange [27].

While additional epidemiological research is needed to confirm the prevalence of HIV, HCV, and HBV among PWID in Kumasi, our mixed methods study identified important needs for health, harm reduction, and evidence-based addiction treatment programming for this population. Addressing stigma related to HIV and addiction within the healthcare and public security sectors will be critical to the success of these effort [28]. The Ghanaian National Police, in collaboration with the global Law Enforcement and HIV Network (LEAHN), have been involved in harm reduction efforts with sex workers and have recognized the unique vulnerability of PWID, presenting an opportunity to engage the police in harm reduction, treatment, and HIV prevention efforts [29].

Our study has several limitations. As is common in studies of highly marginalized groups, we were not able to randomly select participants. For our enumeration, survey, and syringe testing components, we collected data from a convenience sample involving PWID present at Kumasi’s major bases at particular times. Low prevalence in this sample may be attributable to the testing method we used. Instead of testing for blood-borne viruses in saliva or blood taken directly from a person, we tested the contents of the syringes received from each study participant. While this method has been used in other HIV prevalence studies [30–32], it is possible that there was not enough biological material left in syringes. Nevertheless, we believe the findings from this mixed methods study provide useful preliminary information for larger, more representative studies.

## Conclusions

Our findings have compelling implications for the development and implementation of programs and policies that aim to prevent HIV and other blood-borne infections in Ghana and other countries in sub-Saharan Africa [33]. Despite the inclusion of PWID in Ghana’s National HIV/AIDS Strategy, to date, PWID are not prioritized in HIV prevention and treatment efforts. The 2020 Narcotics Control Law offers a critical opportunity to implement and evaluate the effectiveness of evidence-based harm reduction interventions [34]. The results of this research, coupled with those of our 2014 qualitative study, confirm the urgent need for an enabling legal and policy environment necessary to support harm reduction programming for PWID in Ghana.

## Supplementary Information

**Additional file 1**. Socio-demographic characteristics and behaviors of PWID completing surveys at major bases in Kumasi, Ghana (n = 221)

## Data Availability

The datasets used and/or analyzed during the current study are available from the corresponding author on reasonable request.

## Abbreviations

HBV: Hepatitis B virus
HCV: Hepatitis C virus
HIV: Human immunodeficiency virus
PWID: People who inject drugs
