# Supplementary material for: High levels of used syringe use and unsafe sex among people who inject drugs in Kumasi, Ghana: an urgent call for a comprehensive harm reduction approach
Source: Harm Reduct J. 2021 Jun 10;18:62. doi: 10.1186/s12954-021-00510-7 (PMC8194176; doi:10.1186/s12954-021-00510-7)
Supplement: Supplementary file 1 — Additional file 1. Socio-demographic characteristics and behaviors of PWID completing surveys at major bases in Kumasi, Ghana (n = 221) [file 12954_2021_510_MOESM1_ESM.docx]

**Appendix Table X. Characteristics and behaviors of PWID completing surveys at major bases in Kumasi, Ghana (n=221)**

| **Variable** | **Shared syringes**  **(n = 131)**  n (%) | **Did not share syringes**  **(n = 90)** | **Overall**  **(n = 221)** | **p value** | **Unadjusted Odds Ratio** | **95% CI** |
| --- | --- | --- | --- | --- | --- | --- |
|  |  | n (%) | n (%) |  |  |  |
| Age | 34 (31 – 41) | 33 (28 – 40) | 34 (29 - 41) | 0.698^e^ | 1.00 | 0.93 - 1.08 |
| Gender |  |  |  | 0.693 |  |  |
| Male | 122 (93) | 85 (94) | 207 (94) | - | 0.8 | 0.32 - 1.97 |
| Female | 9 (7) | 5 (6) | 14 (6) | - | *Reference* | - |
| Education |  |  |  | 0.132 |  |  |
| Never attended school | 18 (14) | 7 (8) | 25 (11) | - | **2.4** | **1.14 - 5.05** |
| Primary School | 69 (53) | 42 (47) | 111 (50) | - | **1.53** | **1.15 - 2.04** |
| Secondary or higher | 44 (34) | 41 (46) | 85 (38) | - | *Reference* | - |
| Marital Status |  |  |  | 0.684 |  |  |
| Divorced | 38 (29) | 23 (26) | 61 (27) | - | **1.89** | **1.40 - 2.55** |
| Widowed | 3 (2) | 1 (1) | 4 (2) | - | 1.64 | 0.49 - 5.41 |
| Married/Cohabitating | 7 (5) | 8 (9) | 15 (7) | - | **3.43** | **1.26 - 9.34** |
| Never Married | 83 (63) | 58 (64) | 141 (64) | - | *Reference* | - |
| Ethnicity |  |  |  | **0.038** |  |  |
| Akan | 122 (93) | 76 (84) | 198 (90) | - | *Reference* | - |
| Other^a^ | 9 (7) | 14 (16) | 23 (10) | - | **0.40** | **0.18 - 0.88** |
| Religion |  |  |  | 0.448 |  |  |
| Christian | 107 (82) | 77 (86) | 184 (83) | - | *Reference* | - |
| Other^b^ | 24 (18) | 13 (14) | 37 (17) | - | **1.33** | **1.03 - 1.72** |
| Syringe source^c^ |  |  |  |  |  |  |
| Pharmacy | 92 (70) | 70 (78) | 162 (73) | 0.213 | 1.48 | 0.80 - 2.76 |
| Friend | 96 (73) | 63 (70) | 159 (72) | 0.594 | 0.85 | 0.37 - 1.95 |
| Hospital | 2 (2) | 2 (2) | 4 (2) | 1.000^d^ | 1.47 | 0.17 - 12.37 |
| Employment^c^ |  |  |  |  |  |  |
| Laborer | 63 (48) | 38 (42) | 101 (46) | 0.289 | 1.27 | 0.74 - 2.17 |
| Small Business Owner | 15 (11) | 22 (24) | 37 (17) | **0.011** | **0.40** | **0.23 - 0.70** |
| Porter | 69 (53) | 26 (29) | 95 (43) | **<0.001** | 2.74 | 0.63 - 11.96 |
| Sell drugs | 7 (5) | 5 (6) | 12 (5) | 1.000^d^ | 0.96 | 0.38 - 2.42 |
| Sell sex | 6 (5) | 2 (2) | 8 (4) | 0.477^d^ | 2.11 | 0.70 - 6.36 |
| Thief/Robber | 27 (21) | 15 (17) | 42 (19) | 0.463 | 1.3 | 0.44 - 3.82 |
| Income (earn in a day) | 150 (100 – 200) | 150 (80 – 200) | 150 (100 - 200) | 0.864^e^ | 0.99 | 0.99 - 1.00 |
| Number of people live with |  |  |  | 0.534 |  |  |
| 0-3 people | 47 (36) | 36 (40) | 83 (38) | - | *Reference* | - |
| 4 or more people | 84 (64) | 54 (60) | 138 (62) | - | 1.19 | 0.78 - 1.82 |
| Number of drug users you know in your community | 16 (15 – 20) | 15 (10 – 20) | 15 (12 - 20) | **0.016**^e^ | **1.06** | **1.02 - 1.12** |
| Number of places where people inject drugs in your community | 2 (2 – 4) | 3 (2 – 4) | 3 (2 - 4) | **0.034**^e^ | 0.85 | 0.71 - 1.01 |
| Ever tested for HIV |  |  |  | 0.354 |  |  |
| Yes | 43 (33) | 35 (39) | 78 (35) | - | 0.77 | 0.45 - 1.32 |
| No | 88 (67) | 55 (61) | 143 (65) | - | *Reference* | - |
| Weekly Injection Frequency |  |  |  | **<0.001** |  |  |
| 1-20 times | 6 (5) | 20 (22) | 26 (12) | - | *Reference* | - |
| 21 or more | 125 (95) | 70 (78) | 195 (88) | - | **5.95** | **2.84 - 12.48** |
| Number of people injects with |  |  |  | **<0.001** |  |  |
| 0-3 people | 8 (6) | 23 (26) | 31 (14) | - | *Reference* | - |
| 4 or more people | 123 (94) | 67 (74) | 190 (86) | - | **5.28** | **2.89 - 9.65** |
| Stigma experience |  |  |  | 0.376 |  |  |
| Yes | 121 (92) | 80 (89) | 201 (91) | - | *Reference* | - |
| No | 10 (8) | 10 (11) | 20 (9) | - | 0.66 | 0.26 - 1.67 |
| Would use a Syringe Exchange Program if available |  |  |  | **0.002** |  |  |
| Yes | 68 (52) | 28 (31) | 96 (43) | - | **2.39** | **1.37 - 4.16** |
| No | 63 (48) | 62 (39) | 125 (57) | - | *Reference* | - |
| Sex in the last month |  |  |  | 0.906 |  |  |
| Yes | 30 (23) | 20 (22) | 50 (23) | - | 1.04 | 0.64 - 1.70 |
| No | 101 (77) | 70 (78) | 171 (77) | - | *Reference* | - |

1. Category includes Ewe, Mole-Dagbane, Guan, and Ga-Adangbe
2. Category includes Muslim and Traditional
3. Not mutually exclusive
4. Fisher’s Exact Test
5. P-value obtained from the t-test
